# Supplementary material for: Analysis of PPARγ Signaling Activity in Psoriasis
Source: Int J Mol Sci. 2021 Aug 10;22(16):8603. doi: 10.3390/ijms22168603 (PMC8395241; doi:10.3390/ijms22168603)
Supplement: Supplementary file 1 [file ijms-22-08603-s001.zip › Supplemental materials_Analysis of PPARg signaling activity in psoriasis/Pathway models/Models images and html files/Model of PPARG related pathways in psoriasis (short version)/68260.html]

diaminopimelic acid


# Small Molecule diaminopimelic acid

|  |  |
| --- | --- |
| URN | urn:agi-cas:583-93-7 |
| Total Entities | 4 |
| Connectivity | 66 |
| Name | diaminopimelic acid |
| Molecular Weight | 190.197060 |
| XLogP | -5.900000 |

---

|  |  |
| --- | --- |
| ChildConcepts | meso-diaminopimelate |
|  | fructoselysine |
|  | carboxymethyllysine |
|  | furosine |

---

|  |  |
| --- | --- |
| Pathway | NOD-like Receptors |
|  | Dendritic Cell Function in Ulcerative Colitis |
|  | Blau Syndrome |
|  | Model of PPARG signaling in psoriasis |
|  | Model of PPARG related pathways in psoriasis (short version) |
|  | 1\_Insufficient activation of immune response in the middle ear epithelium cells in otitis media |
|  | 2\_Paneth cell dysfunction in Crohn's disease |
|  | 2\_Intestine epithelial cells drive immunological responses in ulcerative colitis |

---

|  |  |
| --- | --- |
| MedScan ID | 1235746 |

---

|  |  |
| --- | --- |
| Alias | (2S,6S)-2,6-diaminoheptanedioate |
|  | 17121-19-6 |
|  | 14289-34-0 |
|  | DIAMINOPIMELIC ACID |
|  | L,L-2,6-diaminoheptanedioic acid |
|  | 2,6-Diaminoheptanedioic acid |
|  | L,L-DAP |
|  | alpha,epsilon diaminopimelic acid |
|  | L,L-A2pm |
|  | 2,6-diamino-Heptanedioic acid |
|  | a,e-Diaminopimelate |
|  | a,a'-Diaminopimelate |
|  | LL-2,6-diaminoheptanedioate |
|  | a,e-Diaminopimelic acid |
|  | 2,6-Diaminoheptanedioate |
|  | (2S,6S)-2,6-diaminoheptanedioic acid |
|  | (S-(R\*,R\*))-2,6-diaminoheptanedioate |
|  | NSC 45225 |
|  | (S-(R\*,R\*))-2,6-diaminoheptanedioic acid |
|  | LL-2,6-diaminoheptanedioic acid |
|  | DIAMINOPIMELATE |
|  | L,L-2,6-diaminoheptanedioate |
|  | EINECS 209-524-6 |
|  | 2,2' diaminopimelic acid |
|  | L,L-diaminopimelate |
|  | 2,6-Diaminopimelic acid |
|  | L,L-2,6-diaminopimelate |
|  | 2,6-diamino-Heptanedioate |
|  | pimelic acid,2,6 diamino |
|  | meso diaminopimelic acid |
|  | 2,6-Diaminopimelate |
|  | 583-93-7 |
|  | (2R,6S)-2,6-diamino-heptanedioate |
|  | (R\*,S\*)-2,6-diamino-Heptanedioic acid |
|  | (R\*,S\*)-2,6-Diaminoheptanedioic acid |
|  | (R\*,S\*)-2,6-diamino-Heptanedioate |
|  | (R\*,S\*)-2,6-Diaminoheptanedioate |
|  | (2R,6S)-2,6-diamino-heptanedioic acid |
|  | D,L-diaminopimelate |
|  | D,L-meso-diaminoheptanedioic acid |
|  | D,L-meso-diaminoheptanedioate |
|  | D,L-DAP |
|  | dl-2,6-Diaminoheptanedioate |
|  | meso-diaminoheptanedioate |
|  | meso-alpha,epsilon-Diaminopimelate |
|  | Heptanedioic acid, 2,6-diamino-, (R\*,S\*)- |
|  | DL-2,6-Diaminopimelic acid |
|  | Heptanedioic acid, 2,6-diamino- |
|  | meso-alpha,alpha'-Diaminopimelate |
|  | dl-2,6-Diaminoheptanedioic acid |
|  | meso-diaminopimelate |
|  | DL-2,6-Diaminopimelate |
|  | meso-alpha,epsilon-Diaminopimelic acid |
|  | M-DAP |
|  | meso-A2pm |
|  | meso-2,6-diamino-Heptanedioic acid |
|  | meso-2,6-diaminoheptanedioic acid |
|  | meso-2,6-diamino-Heptanedioate |
|  | meso-2,6-diaminoheptanedioate |
|  | meso-diaminoheptanedioic acid |

---

|  |  |
| --- | --- |
| CAS ID | 583-93-7 |
|  | 14289-34-0 |

---

|  |  |
| --- | --- |
| Reaxys ID | 1726899 |
|  | 1726900 |
|  | 1726901 |
|  | 1787719 |
|  | 5258985 |
|  | 8218693 |

---

|  |  |
| --- | --- |
| ChEBI ID | 16026 |
|  | 23673 |

---

|  |  |
| --- | --- |
| HMDB ID | HMDB01370 |

---

|  |  |
| --- | --- |
| KEGG ID | C00666 |

---

|  |  |
| --- | --- |
| InChIKey | GMKMEZVLHJARHF-UHFFFAOYSA-N |
|  | GMKMEZVLHJARHF-WHFBIAKZSA-N |

---

|  |  |
| --- | --- |
| Molecular Formula | C7H14N2O4 |

---

|  |  |
| --- | --- |
| PubChem SID | 134978450 |
|  | 135261834 |
|  | 154734 |
|  | 3734107 |

---

|  |  |
| --- | --- |
| PubChem CID | 865 |
|  | 439283 |

---

|  |  |
| --- | --- |
| XLogP-AA | -5.9 |

---

|  |  |
| --- | --- |
| IUPAC Name | 2,6-diaminopimelic acid |
|  | (2S,6S)-2,6-diaminopimelic acid |

---

|  |  |
| --- | --- |
| Rotatable Bond Count | 6 |

---
